# Supplementary figures and images for: Piwi1 is essential for gametogenesis in mollusk Chlamys farreri
Source: PeerJ. 2017 Jun 23;5:e3412. doi: 10.7717/peerj.3412 (PMC5483327; doi:10.7717/peerj.3412)

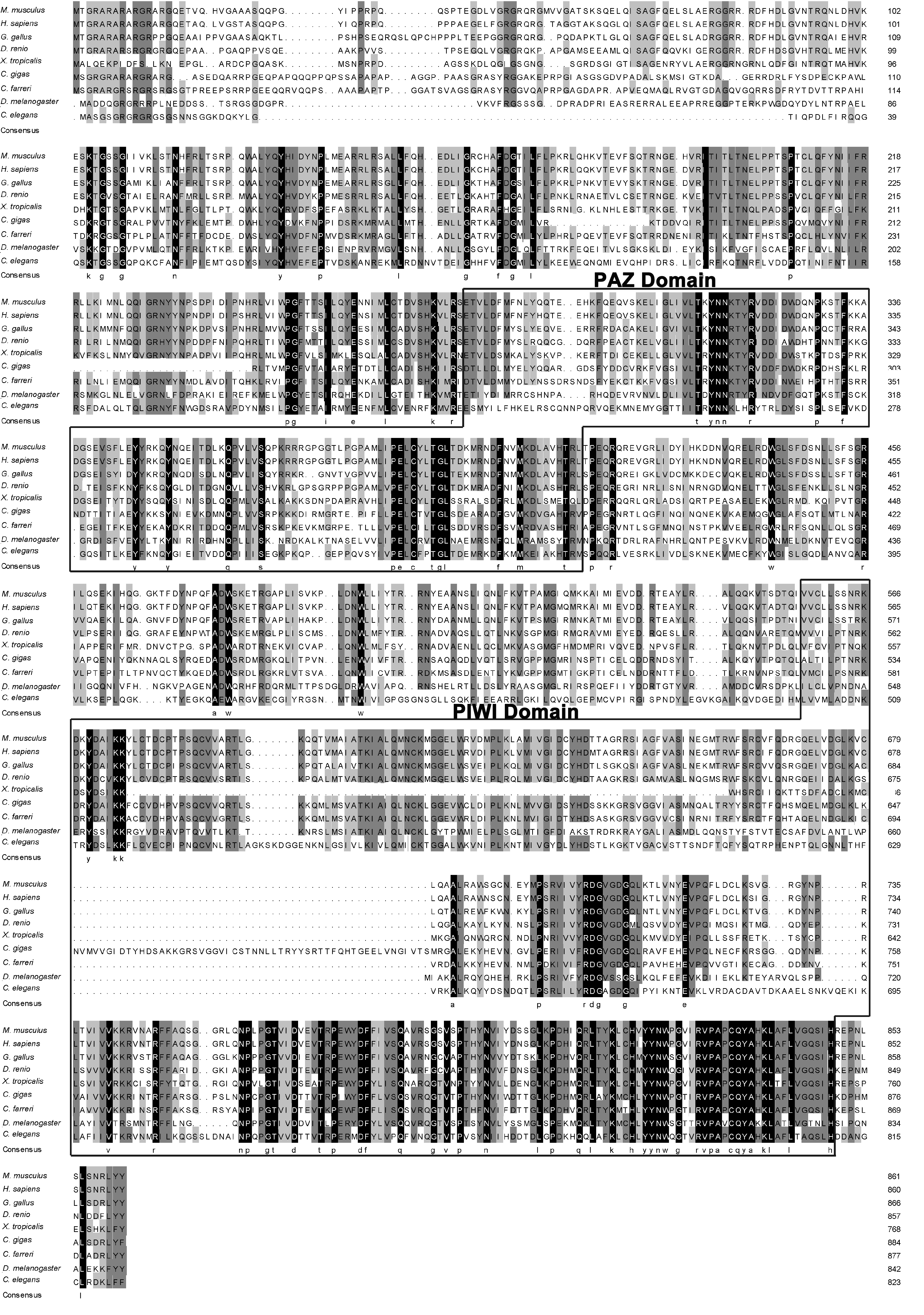

Supplement: Figure S1 — Two boxes show the PAZ (N-terminal side) and Piwi (C-terminal side) domains. The identical and similar residues are highlighted in black and gray, respectively. [file peerj-05-3412-s001.png]
