# Supplementary material for: Piwi1 is essential for gametogenesis in mollusk Chlamys farreri
Source: PeerJ. 2017 Jun 23;5:e3412. doi: 10.7717/peerj.3412 (PMC5483327; doi:10.7717/peerj.3412)
Supplement: Table S1 [file peerj-05-3412-s002.docx]

Supplemental Table1 Quantification of germ cells in five different histological sections of *C. farreri* ovary after RNAi

| group | cell type | section 1 | section 2 | section 3 | section 4 | section 5 |
| --- | --- | --- | --- | --- | --- | --- |
| Blank | ISC | 48 | 50 | 32 | 35 | 39 |
|  | Og | 26 | 28 | 63 | 58 | 45 |
|  | Oc | 30 | 44 | 24 | 19 | 18 |
|  | Moc | 22 | 18 | 24 | 11 | 13 |
| PBS | ISC | 52 | 28 | 20 | 30 | 66 |
|  | Og | 62 | 32 | 35 | 40 | 57 |
|  | Oc | 18 | 16 | 21 | 20 | 18 |
|  | Moc | 14 | 24 | 15 | 19 | 12 |
| dsRNA | ISC | 20 | 16 | 15 | 12 | 19 |
|  | Og | 12 | 16 | 13 | 17 | 11 |
|  | Oc | 18 | 15 | 15 | 10 | 17 |
|  | Moc | 0 | 0 | 0 | 0 | 0 |

ISC, Intragonadal somatic cell; Og, Oogonium; Oc, Oocyte; Moc, Mature oocyte
