# Supplementary material for: Piwi1 is essential for gametogenesis in mollusk Chlamys farreri
Source: PeerJ. 2017 Jun 23;5:e3412. doi: 10.7717/peerj.3412 (PMC5483327; doi:10.7717/peerj.3412)
Supplement: Table S2 [file peerj-05-3412-s003.docx]

Supplemental Table2 Quantification of germ cells in five different histological sections of *C. farreri* testis after RNAi

| group | cell type | section 1 | section 2 | section 3 | section 4 | section 5 |
| --- | --- | --- | --- | --- | --- | --- |
| Blank | Sg | 23 | 29 | 31 | 34 | 40 |
|  | Sc | 145 | 156 | 166 | 176 | 169 |
|  | St | 32 | 39 | 29 | 27 | 21 |
|  | Sz | 23 | 35 | 21 | 33 | 23 |
| PBS | Sg | 22 | 25 | 20 | 30 | 39 |
|  | Sc | 232 | 154 | 187 | 164 | 186 |
|  | St | 33 | 25 | 28 | 39 | 20 |
|  | Sz | 25 | 38 | 14 | 22 | 23 |
| dsRNA | Sg | 33 | 54 | 41 | 43 | 39 |
|  | Sc | 33 | 48 | 62 | 88 | 69 |
|  | St | 15 | 6 | 6 | 9 | 4 |
|  | Sz | 0 | 0 | 0 | 0 | 0 |

Sg, Spermatogonium; Sc, Spermatocyte; St, Spermatid; Sz, Spermatozoon
